# The relationship between ambient temperature and match running performance of elite soccer players

**DOI:** 10.1371/journal.pone.0288494

**Published:** 2023-07-11

**Authors:** Ryland Morgans, Eduard Bezuglov, Dave Rhodes, Jose Teixeira, Toni Modric, Sime Versic, Rocco Di Michele, Rafael Oliveira

**Affiliations:** 1 Football Performance Hub, University of Central Lancashire, Preston, United Kingdom; 2 Department of Sports Medicine and Medical Rehabilitation, Sechenov State Medical University, Moscow, Russia; 3 Research Centre in Sports Sciences, Health and Human Development, Vila Real, Portugal; 4 Departamento de Desporto e Educação Física, Instituto Politécnico de Bragança, Bragança, Portugal; 5 Instituto Politécnico da Guarda, Guarda, Portugal; 6 Faculty of Kinesiology, University of Split, Split, Croatia; 7 Department of Biomedical and Neuromotor Sciences, University of Bologna, Bologna, Italy; 8 Sports Science School of Rio Maior–Polytechnic Institute of Santarém, Rio Maior, Portugal; 9 Life Quality Research Centre, Rio Maior, Portugal; Università degli Studi di Milano: Universita degli Studi di Milano, ITALY

## Abstract

The influence of environmental factors on key physical parameters of soccer players during competitive match-play have been widely investigated in the literature, although little is known on the effects of sub-zero ambient temperatures on the performance of adult elite soccer players during competitive matches. The aim of this study was to assess how the teams’ match running performance indicators are related to low ambient temperature during competitive matches in the Russian Premier League. A total of 1142 matches played during the 2016/2017 to 2020/2021 seasons were examined. Linear mixed models were used to assess the relationships between changes in ambient temperature at the start of the match and changes in selected team physical performance variables, including total, running (4.0 to 5.5 m/s), high-speed running (5.5 to 7.0 m/s) and sprint (> 7.0 m/s) distances covered. The total, running and high-speed running distances showed no significant differences across temperatures up to 10°C, while these showed small to large decreases at 11 to 20°C and especially in the >20°C ranges. On the contrary, sprint distance was significantly lower at temperature of -5°C or less compared to higher temperature ranges. At sub-zero temperatures, every 1°C lower reduced team sprint distance by 19.2 m (about 1.6%). The present findings show that a low ambient temperature is negatively related to physical match performance behavior of elite soccer players, notably associated with a reduced total sprint distance.

## Introduction

Soccer is a worldwide sport that is played in differing environmental conditions varying from extreme heat to severely cold temperatures. The physical demands that are required on players can vary significantly as a result of the environmental conditions in which soccer matches are played [1–3]. Over the course of a soccer season, environment conditions can change significantly from warm summer months to cold winter months within most European soccer leagues. These changes may dictate important considerations for the physical preparation of players over the course of a soccer season in relation to the specific environmental conditions experienced locally.

Much of the previous research that has been undertaken into the effects of environmental conditions on soccer performance has focused on the impact of hot environments and exposure to altitude [4]. While this may be of significant importance in certain areas of the world, it would appear that it is also important to investigate the impact of extremely cold temperatures which are more commonly seen during the winter months of many European leagues. There is scant previous research in this area, however, some studies have assessed the effects of cold environments on physical performance during soccer match-play [5]. For instance, this study showed greater distances covered by midfielders at high-intensity running (>19.8 km/h), between the 30-45-minute period, in temperatures ≤5°C versus ≥21°C. However, total distance covered was unaffected in colder conditions, although this finding could not be confirmed across all positions as defenders and strikers were not assessed [5].

Assessment of the effects of different temperatures on the physiological response to submaximal exercise in soccer players shows that exercise capacity is reduced in cool environments when compared with moderate conditions [6]. This was evident by changes in players’ heart rate responses and ventilation rates in cool environments when cycling for 20 minutes at 60% maximum oxygen uptake and until reaching exhaustion in three different environmental conditions (10, 22, 35°C) [6]. Furthermore, Armstrong [7] stated that exercising in cold environments has various effects on physiological processes such as an increase in muscle glycogen utilization, a reduction in aerobic capacity, and a reduction in muscular strength and power.

More specifically, Carling *et al*. [5] investigated the effect of cold temperature on physical activity profiles in matches over four seasons in the French Ligue 1. The authors reported that generally physical outputs were unaffected by changes in environmental temperatures in the matches analyzed. However, the lowest match temperature involved in this study were ≤5°C. This temperature accounts for a large range of temperatures which may significantly affect physical match performance. Although, further research may be required to assess the differences in the effect on physical match performance of very cold and freezing temperatures (<5°C).

Therefore, the aim of this study was to investigate the relationships between ambient temperatures and physical match activity of elite soccer players during official competitive matches in the Russian Premier League, where matches are often played in a cold environment, especially during autumn and winter months. Given the different findings of the previous studies about the lower capacity of exercising in a cold environment on physical performance [6, 7], and at the same time that midfielders run higher distances at a speed > 19.8 km/h [5], it was hypothesized that playing at very low temperatures would impact physical match behavior when compared with warmer environmental conditions, specially at speeds < 19.8 km/h.

## Materials and methods

### Experimental design

An observational design to assess the impact of ambient temperature on soccer match physical performance in official matches played in the Russian Premier League over a period of five competitive seasons was employed.

### Match sample

The ambient temperature at the start of the match, and a selection of the team’s physical match performance variables, were assessed for all official matches played in the Russian Premier League during five consecutive seasons, 2016/2017 to 2020/2021. Due to some missing temperature and physical performance data, and the poor quality of some match data due to environmental or other reasons, a total of 1146 out of 1200 matches were examined. For every match, data for the home and away team were examined, resulting in a total of 2292 team performance data points, from a total of 24 different teams. Ethical approval for the study was granted by the Ethics Committee of Sechenov University (N06-21 dated 04/07/2021). The study was performed in accordance with the Helsinki Declaration principles. All professional players and the Russian Premier League agreed to the collection of match performance data and for research purposes.

### Data collection

League match data across each season was recorded and analyzed via a two-camera Optical Tracking System (InStat, Moscow, Russia) to report physical performance data. The matches were filmed using two full HD, static cameras positioned on the centre line of the field, not less than 3-metres from the field and 7-metres in height. A consistent 25Hz format was provided. Data were linearly interpolated to 50Hz, smoothed using a 5-point moving average and then down-sampled to 10Hz. The reliability and validity of InStat have been demonstrated by assessing velocity and position data collected during soccer-specific exercises in comparison with a reference stereophotogrammetric system (Vicon Motion Systems Ltd., Oxford, UK). These assessments are included in the official FIFA test protocol for Electronic and Performance Tracking Systems document, stating that the system has passed [8]. The InStat Analysis Software System was used to measure and analyze physical performance. InStat provided written permission to allow all match data to be used for research purposes.

The following distances, describing the whole team physical match performance, were assessed: total distance covered by all team players (m); running distance covered by all team players (m; total distance covered 4.0 to 5.5 m/s); high-speed running (HSR) distance covered by all team players (m; total distance covered 5.5 to 7.0 m/s); and sprint distance covered by all team players (m; total distance covered >7.0 m/s). These variables were selected based on previous studies [9, 10] to facilitate comparisons.

The League Development Department gathered temperature data on all official matches during the 2015 to 2021 period from the official Hydrometeorological Center. Meteorological data were retrieved from the meteorological stations located nearest the stadiums, available from the Russian Weather Service (http://aisori-m.meteo.ru/waisori/) and Ogimet (https://www.ogimet.com/synops.phtml.en) databases. As far as data from the stadiums were concerned, meteorological data were thus taken per hour during the match-day and one hour prior to kick-off, measured from the centre of the field, as stated by League requirements. Data on air temperature during matches were retrieved from the stadiums’ meteorological stations and reported by the official match delegate. Two experts retrospectively examined and verified the official Hydrometeorological Center data by comparisons with the League delegate reports. All matches were classified into six groups depending on the ambient temperature at the start of the match: <-5°C; -4 to 0°C; 1 to 5°C; 6 to 10°C; 11 to 20°C; >20°C. Ranges for temperatures higher than 5°C were set according to Carling *et al*. [5], while two additional ranges (-4 to 0°C and 1 to 5°C) were added for temperatures up to 5°C. The lowest temperature threshold (<-5°C) was set according to Link and Weber [11]. All physical match performance and match temperature data were analysed by the same experienced investigator to ensure inter-observer reliability was not a limitation of the study.

### Statistical analysis

The statistical analysis was conducted using the software R, version 4.2.0 (R Foundation for Statistical Computing, Vienna, Austria). Data are presented as mean ± standard deviation. Linear mixed models with random intercept on team IDs were used to compare the examined physical performance variables across temperature ranges. When there was a significant (p<0.05) difference between temperature ranges, Tukey’s tests were used to determine which ranges differed. The estimated differences were standardized by dividing them by the estimated between-team standard deviation to determine the effect size (ES). Absolute ES values were evaluated as <0.2, trivial; 0.2–0.6, small; 0.6–1.2, moderate; 1.2–2.0, large; 2.0–4.0, very large; >4.0 extremely large [12].

The effect of low temperature on physical match performance was further explored by taking temperature as a continuous variable. Linear mixed models with random intercept on team IDs were used to examine the effect of 1°C ambient temperature decrease on physical performance variables. For this analysis, only matches started at ambient temperature < 0°C were included. Effect sizes were calculated as Cohen’s d from the coefficients of linear mixed models coefficient with the lme.dscore function from package EMAtools [13], and interpreted as very small (<0.2), small (0.2–0.5), medium (0.5–0.8), large (>0.8) [14].

For all models, it was ensured that the assumptions of linearity, homoscedasticity and normality of residuals were met by visually inspecting histograms of residuals and plots of residuals vs. fitted values. For all analyses, statistical significance was set at p<0.05.

## Results

In the examined matches, the mean ambient temperature at the start of the match was 11.7 ± 10.2°C.

Table 1 presents the mean values for the examined physical performance variables in each temperature range, while Table 2 displays the standardized differences (ES). The total distance was similar from the <-5°C to the 6 to 10°C ranges, while it decreased in the 11 to 20°C range with significant small differences vs. all the lower temperature ranges. In the >20°C range, the total distance shows a further increase, with significant moderate differences vs. all the lower temperature ranges. A similar trend was observed for running distance, with no significant differences between ranges from <-5°C to 6 to 10°C, a decrease in the 11 to 20°C range, with significant moderate differences vs. all the lower temperature ranges and a marked decrease in the >20°C range, with significant moderate to large differences vs. all lower temperature ranges (Tables 1 and 2). The HSR distance was slightly though not significantly lower in the <-5°C range when compared to temperature ranges from -4 to 0°C to 6 to 10°C. In the 11 to 20°C range, HSR was significantly lower than in the -4 to 0°C, 1 to 5°C, and 6 to 10°C ranges, with small differences. In the >20°C range, HSR distance was significantly lower than all lower temperature ranges, with moderate to large differences. Sprint distance was similar across ranges from -4 to 0°C to 11 to 20°C, while it was significantly lower in the <-5°C range when compared to all higher temperature ranges except >20°C with moderate differences, and in the >20°C range when compared to all lower temperature ranges except <-5°C, with differences ranging from small to moderate (Tables 1 and 2).

**Table 1:** Mean ± SD values, with 95% Confidence Intervals (CI), for physical match performance variables across temperature ranges.

		<-5°C	-4 to 0°C	1 to 5°C	6 to 10°C	11 to 20°C	>20°C
		(n = 134)	(n = 222)	(n = 322)	(n = 372)	(n = 726)	(n = 516)
Total distance (km)	Mean ±SD	114.9^d^^e^ ± 4.8	114.3^d^^e^ ± 5.1	114.5^d^^e^ ± 4.7	114.6^d^^e^ ± 4.6	113.7^e^ ± 4.8	112.3 ± 5.5
	95% CI	114.1–115.8	113.6–114.9	114.0–115.0	114.1–115.1	113.4–114.0	112.8–111.9
Running distance (km)	Mean ±SD	20.0^d^^e^ ± 1.8	20.0^d^^e^ ± 2.0	20.1^d^^e^ ± 1.8	20.0^d^^e^ ± 2.0	19.3^e^ ± 1.8	18.3 ± 1.8
	95% CI	19.7–20.3	19.7–20.3	19.9–20.3	19.8–20.2	19.2–19.5	18.2–18.5
High-speed distance (km)	Mean ±SD	7.7^e^ ± 0.9	7.9^d^^e^ ± 0.9	7.9^d^^e^ ± 0.9	7.9^d^^e^ ± 1.0	7.7^e^ ± 0.9	7.2 ± 0.9
	95% CI	7.5–7.8	7.8–8.0	7.8–8.0	7.8–8.0	7.6–7.7	7.1–7.3
Sprint distance (km)	Mean ±SD	1.1^a^^b^^c^^d^ ± 0.4	1.2^e^ ± 0.4	1.2^e^ ± 0.3	1.3^e^ ± 0.4	1.3^e^ ± 0.4	1.1 ± 0.4
	95% CI	1.0–1.1	1.2–1.3	1.2–1.3	1.2–1.3	1.2–1.3	1.1–1.2

^a^ denotes a significant difference vs. -4 to 0°C

^b^ denotes a significant difference vs. 1 to 5°C

^c^ denotes a significant difference vs. 6 to 10°C

^d^ denotes a significant difference vs. 11 to 20°C

^e^ denotes a significant difference vs. >20°C. Number of data is referred to team performance data points

**Table 2:** Standardized differences (ES), with 95% Confidence Intervals (CI), between temperature ranges (values of temperature categories in columns minus values of temperature categories in rows).

		<-5°C	-4 to 0°C	1 to 5°C	6 to 10°C	11 to 20°C
Total distance	-4 to 0°C	0.16 (-0.27 to 0.59)				
	1 to 5°C	0.12 (-0.29 to 0.59)	-0.04 (-0.38 to 0.30)			
	6 to 10°C	0.08 (-0.31 to 0.48)	-0.07 (-0.41 to 0.27)	-0.03 (-0.33 to 0.27)		
	11 to 20°C	0.55* (0.18 to 0.92)	0.39* (0.09 to 0.70)	0.43* (0.17 to 0.70)	0.47* (0.21 to 0.72)	
	>20°C	1.18* (0.80 to 1.57)	1.02* (0.70 to 1.35)	1.06* (0.78 to 1.35)	1.10* (0.83 to 1.37)	0.63* (0.40 to 0.86)
Running distance	-4 to 0°C	0.11 (-0.32 to 0.53)				
	1 to 5°C	0.01 (-0.39 to 0.41)	-0.10 (-0.43 to 0.24)			
	6 to 10°C	0.10 (-0.29 to 0.49)	-0.01 (-0.34 to 0.32)	0.09 (-0.21 to 0.38)		
	11 to 20°C	0.72* (0.35 to 1.08)	0.61* (0.32 to 0.91)	0.71* (0.45 to 0.97)	0.62* (0.37 to 0.87)	
	>20°C	1.73* (1.36 to 2.11)	1.63* (1.31 to 1.94)	1.72* (1.45 to 2.00)	1.64* (1.37 to 1.90)	1.01* (0.79 to 1.24)
High-speed distance	-4 to 0°C	-0.28 (-0.90 to 0.34)				
	1 to 5°C	-0.33 (-0.91 to 0.25)	-0.05 (-0.55 to 0.44)			
	6 to 10°C	-0.34 (-0.91 to 0.23)	-0.06 (-0.55 to 0.42)	-0.01 (-0.44 to 0.42)		
	11 to 20°C	0.21 (-0.33 to 0.74)	0.48* (0.05 to 0.92)	0.54* (0.16 to 0.92)	0.55* (0.18 to 0.91)	
	>20°C	1.31* (0.76 to 1.86)	1.59* (1.13 to 2.05)	1.64* (1.24 to 2.05)	1.65* (1.27 to 2.04)	1.11* (0.78 to 1.43)
Sprint distance	-4 to 0°C	-0.70* (-1.23 to -0.18)				
	1 to 5°C	-0.85* (-1.34 to -0.35)	-0.15 (-0.57 to 0.27)			
	6 to 10°C	-0.93* (-1.42 to -0.45)	0.23 (-0.63 to 0.18)	-0.08 (-0.45 to 0.29)		
	11 to 20°C	-0.84* (-1.29 to -0.39)	-0.13 (-0.50 to 0.24)	0.02 (-0.31 to 0.33)	0.10 (-0.21 to 0.40)	
	>20°C	0.18 (-0.29 to 0.65)	0.52* (0.13 to 0.92)	0.67* (0.32 to 1.06)	0.75* (0.42 to 1.08)	0.66* (0.38 to 0.94)

* denotes a significant difference (p<0.05).

The linear mixed model analysis performed using ambient temperature as a quantitative independent variable including matches when temperature was equal to zero or lower, when examining the estimated fixed effect coefficients, revealed small (d = 0.21–0.23) though non-significant (p >0.05) increases of total distance and running distance for a 1°C decrease of ambient temperature. High-speed running distance showed a very small (d = 0.07) yet non-significant decrease with decreasing temperature. Conversely, there was a significant (p<0.01) decrease of 19.2 m (approximately 1.6%) for every 1°C decrease of ambient temperature (d = 0.48, small).

## Discussion

The present study assessed the relationships between low ambient temperature and physical match behavior in elite soccer players. To this aim, competitive matches played across five consecutive seasons in the Russian Premier League were examined. Although there is a mid-season winter break (mid-December to end-February) during the extremely cold months of the year in this League, there is still a significant number of matches played under cold or very cold conditions, mainly in the weeks just before or after the winter break. Thus, an evaluation into how low temperatures may impact the physical match output of soccer players in a real-world setting is of significant interest to the support staff tasked with maintaining the well-being of players.

Together, our results show that a very cold (<-5°C) ambient temperature at the start of the match has no impact on total distance covered or distance when running up to 5.5 m/s, while there is a trend suggesting a slight reduction of HSR distance (5.5 to 7.0 m/s), and an evident decrease in sprint distance (>7.0 m/s). On average, in matches played at a temperature of -5°C or less, team sprint distance was approximately 10–15% lower than in matches played at less cold or warmer temperatures (Table 1). Moreover, with a mixed-effects regression analysis, a moderate but relevant decrease of approximately 19.2 m (1.6%) of sprint distance covered by the team for every 1°C decrease of ambient temperature when the temperature was 0°C or less was reported. Thus, players performed less sprinting at low temperatures. Overall, the results support the study hypothesis that physical match performance is negatively affected by playing at low temperatures, though the impact of cold environment was evident, among the examined variables, distances covered at high- to maximal intensity were notable.

To date, a number of previous studies have investigated how environmental conditions, including ambient temperature, affects physical performance in soccer players [1, 3, 5, 11, 15–18]. There is some evidence that total distance covered during a soccer match, as well as HSR distance, are reduced in hot environments compared to neutral temperature conditions [1, 11, 16]. Though the present study mainly focused on the impact of low temperatures, the sample included all available matches from the examined Russian Premier League seasons, thus allowing a comparison between physical match performance across all temperature conditions. Consistently with previous studies [1, 11, 17], it was observed that total distance covered, as well as running and HSR distances, were reduced in the 11 to 20°C and more evidently in the >20°C condition than in conditions where the temperature was between -4 and 20°C. Sprint distance also showed a decrease when the temperature at the start of the match was 20°C or higher when compared to lower temperature ranges (Table 1).

Currently, scant literature is available examining the impact of low temperatures on physical match performance in elite soccer. Carling *et al*. [5], analyzed physical match performance in players from a French Ligue 1 team, and showed no detrimental effect of low temperature on distances covered in the 0.0–4.0, 4.0–5.5, and >5.5 m/s speed ranges. However, the authors employed <5°C as the lower temperature condition. Our findings showed no substantial effect of temperature in the 1 to 5°C range, on physical match outcomes, while the effects of low temperature became more evident at -5°C or less. Therefore, the present results are consistent with those of Carling and colleagues [5]. Link and Weber [11] examined the effects of ambient temperature on total distance covered by players from 38 teams from the top two German leagues, collected across 1211 league matches. Those authors used a lower temperature range of <-5°C, as in the present study, and also reported no substantial differences in the total distance covered between matches starting at a temperature of -5°C or less and matches played in the -4° to 13°C and 14 to 27°C temperature ranges. This observation is also consistent with our finding that total distance covered is unaffected by low temperatures (Tables 1 and 2).

A hypothesis that may partly explain why sprint distance may decrease during soccer matches played in very cold (<-5°C) temperatures is that of a reduced sprinting capacity, that may occur due to the negative effects of low temperature on muscle function and power production. Indeed, it has been shown that speed, as well as agility and lower-limb power, is impaired immediately after the application of different cryotherapy modalities [18–20]. To our knowledge, only the study of Carlson *et al*. [21] investigated the effects of whole-body exposure to low temperature on the outcomes of lower-limb power, agility and sprint tests. Reduced vertical jump and agility performance was observed, although unaffected sprinting performance, in recreational athletes, after a 15-minute cool (6.1°C) exposure vs. a thermoneutral (17.2°C) environment was reported [21]. However, the temperature administered in this study for the cool environment is higher than the lower ranges of ambient temperature in the present sample of soccer matches (<-5°C). A colder temperature (-14°C) was utilized by Wiggen and colleagues [22] to examine the effect of cold exposure on double poles sprint performance in cross-country skiers. The authors reported lower performance in terms of power output at 14°C vs. 6°C temperatures. Such evidence supports the assumption that very cold ambient temperature can impair sprinting performance in soccer players, however future studies are warranted to further explore the effect of low ambient temperature on sprinting performance in training and competitive match-play in players from different leagues.

A further factor that may potentially have an impact on reduced sprint distance at low temperatures is possibly related to playing surface conditions. At sub-zero temperatures, the playing turf may be frozen or partly frozen and slippery, decreasing stability and traction, increasing the player ground contact and surface interaction, and therefore making it more difficult for players to execute maximal or near-maximal actions, including sprints, than in normal conditions. Additionally, the team playing strategy may be altered due to elements such as unpredictable ball roll, bounce and ball-speed, leading players to execute more shorter passes and thus reducing the number of longer, forward passes and subsequent physical actions involving long (>30 m) sprinting. In this respect, in future investigations it would be practically interesting to assess how the technical and tactical performance is affected when matches are performed in cold or very cold conditions.

### Limitations

Despite the findings, several limitations of this study were identified. Due to data availability, only ambient temperature at the start of the match was examined as an indicator of environmental conditions, while other atmospheric conditions such as atmospheric pressure, wind, wind child, humidity, heat index, wet-bulb globe temperature, precipitation and cloudiness that may also influence physical performance were not considered. However, depending on other factors such as kick-off time, ambient temperature could to some extent increase or decrease throughout the duration of approximately 2-hours of a soccer match, especially if the kick-off time is in the evening hours. A perspective for future studies is therefore to consider the average ambient temperature during the match. Furthermore, our study lacked data related to other situational variables such as match location, that may also potentially modulate the impact of low temperatures on physical match performance, or others such as match result and quality of the teams that could also cause different running-based results considering the different scenario of the analyzed team. For such reasons, it is recommended to consider those variables in future studies.

## Conclusion

The present study has shown that sub-zero ambient temperatures, especially when equal to or lower than -5°C, are related to changes in the match running performance behavior in elite soccer players. A novel finding of our study is that low temperatures are associated with reduced sprint performance. The present results may be of real practical interest to coaching staff who are responsible for improving and maintaining the health and well-being of soccer players that regularly play in cold or very cold conditions.
